# Adverse Health Effects in Women Farmers Indirectly Exposed to Pesticides

**DOI:** 10.3390/ijerph18115909

**Published:** 2021-05-31

**Authors:** Jose Martin-Reina, Alfredo G. Casanova, Bouchra Dahiri, Isaías Fernández, Ana Fernández-Palacín, Juan Bautista, Ana I. Morales, Isabel Moreno

**Affiliations:** 1 Department of Nutrition, Food Chemistry and Toxicology, Faculty of Pharmacy, University of Sevilla, 41012 Sevilla, Spain; josemartinreina@yahoo.es (J.M.-R.); boudahkha@gmail.com (B.D.); imoreno@us.es (I.M.); 2 Toxicology Unit, Department of Physiology and Pharmacology, Institute of Biomedical Research of Salamanca (IBSAL), University of Salamanca (USAL), 37007 Salamanca, Spain; alfredogcp@usal.es; 3 Estepa Clinical Management Unit, Andalusian Health Service, C/Médico Antonio Vilches s/n, 41560 Sevilla, Spain; isaiasm.fernandez.sspa@juntadeandalucia.es; 4 Area of Preventive Medicine and Public Health, Facultad de Medicina, Avda. Dr. Fedriani, s/n, 41009 Sevilla, Spain; afp@us.es; 5 Department of Biochemistry and Molecular Biology, Faculty of Pharmacy, University of Sevilla, 41012 Sevilla, Spain; jdbaut@us.es

**Keywords:** cholinesterase, oxidative stress, early kidney damage, pesticides, women farmers

## Abstract

Farmers are among the most vulnerable populations because of the exposure to low levels of pesticides. Acetylcholinesterase and butyrylcholinesterase activities are considered as biomarkers of pesticides poisoning. However, biomarkers of oxidative stress are also playing an important role in toxicity of these contaminants. Further, increased activities of gamma-glutamyltransferase, alanine aminotransferase, urea and creatinine have been linked with hepatic and nephrotoxic cell damage, respectively. The aim of this study was to ascertain if the indirect exposure to pesticides leads to some biochemical parameter changes. Thus, cholinesterase activities, oxidative stress status (lipid and protein oxidation), hepatic function (AST and ALT levels), hormonal function (TSH, T4, FSH, LH and AMH), renal function (serum creatinine and urea), as well as possible subclinical kidney damage (urinary proteins and biomarkers of early kidney damage) were evaluated in farmer women who collect fruits and vegetables comparing with a group of women non-occupational exposed to pesticides but living in the same rural environment. Samples were taken periodically along one year to relate the observed effects to a chronic exposure. Our main results showed for the first time a subclinical kidney damage in a rural setting with indirect chronic exposure to pesticides.

## 1. Introduction

Agrochemicals, including pesticides, are extensively used in agriculture practices to kill pests that harm crops, enhancing agricultural productivity. These chemicals are potentially toxic to some organisms, including humans, and need to be safely used and properly disposed [1]. However, due to their indiscriminate use in a number of applications there is a high risk of exposure to these pesticides through occupational and non-occupational settings [2]. Agricultural workers are among the most vulnerable working populations due to social and cultural risk factors frequently associated with their ethnicity, immigration status, social class and rural location. In addition, these potential risks factors can be exacerbated by occupational hazards associated with agricultural work [3]. The three major routes of pesticide entry into a farmer’s organism are inhalation, ingestion and dermal absorption. Exposure to low-levels of pesticides is known to produce a variety of biochemical changes, some of which may be responsible for the adverse biological effects reported in humans [4]. Furthermore, in a real-life situation it is commonly observed the use of multiple pesticides which are related to higher incidence of pesticide poisonings and deaths. The interaction of two or more chemicals may exhibit synergistic effects that could potentially cause damage to various organ systems of the body. The type and severity of adverse health effects of pesticides are determined by the individual chemical category, the dose and the duration of exposure, the exposure route and the use of personal protective equipment (PPE) [3,5,6].

Chronic and acute exposures to pesticides are assessed by the levels of their biomarkers, which are cholinesterase enzymes, acetylcholinesterase activity (AChE) in red bloods cells and butyrylcholinesterase activity (BChE) in plasma. BChE is reduced more rapidly and intensely than AChE, reflecting acute exposure to toxic agents. AChE is, in fact, a more accurate biomarker of chronic and low-intensity exposures [7,8]. Both enzymes levels in blood are considered as biomarkers of the exposure to organophosphate and carbamate pesticides [1]. However, oxidative stress plays an important role in toxicity of a wider range of pesticides including pyrethroid and carbamate insecticides and herbicides as glyphosate [7,9]. In addition to increasing the production of free radicals, exposure to these pesticides can also affect antioxidant capacity and defense mechanisms, as well as increase lipid peroxidation. The by-product most often measured is malondialdehyde (MDA), one of the main lipid hydroperoxides produced by the peroxide degradation of polyunsaturated fatty acids [8,10]. Thus, oxidative stress has been proposed as a mechanism linking exposure to pesticides to increased risk for the development of diseases such as cancer, renal and neurodegenerative diseases [11,12,13] and reproductive disorders [14,15,16]. Further, alterations in the hematological parameters such as decrease size of red blood cells, higher platelets and white blood cell (WBC) counts and increased activities of gamma-glutamyltransferase (GGT), alanine aminotransferase (ALT), alkaline phosphatase (ALP), bilirubin has been shown to be linked with hepatic cell damage in human occupationally exposed to the pesticide [4,17,18]. Conversely, nephrotoxic changes as evidenced by elevated levels of plasma urea, uric acids and creatinine in workers occupationally exposed to pesticides have also been reported [2,19]. Other hematological parameters such as biomarkers of the thyroid function have been less studied but there is some increasing evidence that occupational exposure to agricultural pesticides may affect thyroid function. Thus, it has been observed a significant decreased in serum levels of thyroid-stimulating hormone (TSH) and significant increases in free thyroxine (FT4) and total triiodothyroxine (TT3) in farmers from Brazil [2,20].

It is necessary to delve into the evaluation of the impact of occupational exposure to agrochemicals in order to estimate the risk and develop effective strategies to prevent these health problems. The increased use of pesticides other than organophosphates makes it necessary to look for new biomarkers beyond the classics used in agricultural health surveillance protocols. Most of the epidemiological studies about pesticides exposition are focused on agricultural workers who handle and apply the pesticides on the field (direct exposure) but there are hardly any studies about indirect exposition of workers who collect the fruit and vegetables after the waiting period recommended by pesticide manufacturers before the re-entry on the field. They are chronically exposed indirectly to pesticides trace levels [3]. Alterations in fertility, procreation and development of offspring caused by chronic pesticides exposure made women one of the most vulnerable populations; however, there are only few epidemiologic studies about it. 

In Spain, the health evaluation of workers is mandatory as public policy, and it is recommended once a year. Specifically, the health of pesticide applicators is evaluated only by general clinical tests, such as markers of hepatic function (AST and ALT) and cholinesterase activities, in spite of being only organophosphate and carbamates exposure biomarkers. However, these tests are not mandatory for farmers who collect the fruits and vegetables who also are indirectly exposed to pesticides in their work. The few published Spanish epidemiologic studies are focused on greenhouse pesticides sprayers [5,6,10]. Taking these premises in consideration, this study was conducted with the aim of ascertaining if also the indirect exposure to pesticides leads to some biochemical parameter changes. Thus, cholinesterase activities, oxidative stress status (lipid peroxidation and protein oxidation), hepatic function (AST and ALT levels), hormonal function (TSH, T4, FSH, LH and AMH) and renal function (serum creatinine and urea), as well as possible subclinical kidney damage (urinary proteins and biomarkers of early kidney damage) were evaluated in farmer women who collect fruits and vegetables comparing with a group of women non-occupational exposed to pesticides but living in the same rural environment. 

To our best knowledge, no study has been done on these all parameters together by taking into account this indirectly exposed population of harvest farmer women.

## 2. Materials and Methods

### 2.1. Chemicals

All chemicals, including acetylthiocholine iodide, butyrylthiocholine, thiobarbituric acid and 2,4-dinitrophenylhydrazine were obtained from Sigma-Aldrich (Madrid, Spain).

### 2.2. Study Population

A longitudinal study was conducted on a cohort of 39 women in fertile age from Marinaleda (Sevilla, Spain). Marinaleda is a small town with an area of 24.8 km^2^ and a total population of 2665 inhabitants. Of the total population, 65.2% are between 20 and 65 years of age, of which 1299 are women, representing 49% of the population. Its political and social organization makes it unique and suitable for the achievement of this study. The economic activity of this town is based on agriculture where jobs are distributed among the male and female population being women in charge to collect the fruits and vegetables in the field or work in the factory where these fruits and vegetables are canned. The selection of participants was non probabilistic in a snowball sampling. A final sample of 39 women (age between 18 and 45 years) were asked to be part of this longitudinal study. In this case, 22 of them, directly involved in collection of fruits and vegetables in the field, were classified as farmers and the 17 remaining participants who work in the canned factory and live in the same rural environment were classified as non-occupational exposed (NOE) group. The study was carried out over a year (from October 2017 to October 2018), during which samples were collected every three months in order to study the effects of the chronic indirect exposure to pesticides. The chronogram of sampling, the crops collected at this time and the pesticides most used for these crops are listed in Table 1. Women with a clinical diagnosis of chronic diseases were excluded. A preliminary questionnaire was specifically designed for the study to record the personal and occupational information of all the participants. Ethical clearance was obtained from the Coordinating Committee of Ethics of Biomedical Research of Andalusia (Spain) (code: 0231-N-17/6 February 2017) and was in agreement with the Declaration of Helsinki for International Health Research. Written informed consent was also obtained from all the participants after being informed about the objectives of the study and the right to drop out of the study at any time.

### 2.3. Sample Collection

Blood samples (≈15 mL of venous blood) were obtained from all the participants every three months along the study period. The blood samples were kept in heparinized and nonheparinized tubes and kept in an ice box until transported to the laboratory (just a few hours after the samples collection). Both plasma and serum were separated from the blood and kept refrigerated until their use. The whole blood samples were used for the hematological test while plasma and serum samples were used for the biochemical analysis.

### 2.4. Assay of Cholinesterase’s Activities

Red blood cells AChE activity was measured as described by Ellman et al. (1961) [21] and adapted for microplates, as described by Guimarães et al. (2007) [22]. Briefly, acetylthiocholine iodide (AcSCh) 9 mM was used as substrate and 5,5′-dithio-bis (2-nitrobenzoic) 0.75 mM (DTNB) acid as chromogen. The optical density at 415 nm was measured each 30 s for 3 min using an ELISA plate reader (Synergy HTX, BIO-TEK, Winooski, Vermont, U.S.A). Enzyme activity is expressed as U/L.

Plasma cholinesterase (BChE) was determined by measuring the rate of hydrolysis of butyrylthiocholine (BuSCh) 24 mM instead of AcSCh and 0.5 mM DTNB as chromogen according to the same method mentioned above. Enzyme activity is expressed as U/L.

### 2.5. Hematological and Biochemical Analysis

The following hematological parameters were determined using a hematology analyzer (Sysmex XN-1000, Norderstedt, Germany) in whole blood collected in heparinized tubes: red blood parameters (number of red blood cells, ×10^6^/µL), hemoglobin (g/dL), hematocrit (%), white blood parameters [total number of leukocytes, ×10^3^/µL, total number (×10^3^/µL) and percentage of neutrophils, lymphocytes, eosinophils, monocytes and basophils] and platelet count (×10^3^/µL). 

Biochemical parameters were measured on fresh serum samples using a clinical analyzer (Vital Scientific-Selectra XL, Spankeren, The Netherlands) following standard procedures for clinical biochemistry. The measured parameters included: glucose, total proteins (g/dL), lipid profile: total cholesterol, high-density lipoprotein (HDL), low-density lipoprotein (LDL), triglycerides; liver function test: alanine aminotransferase (ALT), aspartate aminotransferase (AST), alkaline phosphatase (ALP) and lactate dehydrogenase (LDH) (units for all these tests are U/L); and renal function test: creatinine (mg/dL) and urea (mg/dL). 

### 2.6. Hormonal Analysis

Thyroid hormone levels were measured on fresh serum samples by paramagnetic particles, chemiluminescent immunoassay using a CL-1000i analyzer (Mindray, Shenzhen, China). The measured hormones were levels of thyroid-stimulating hormone (TSH) (µUI/mL) and free thyroxine (FT4) (ng/dL). The reference values were 0.270–5.500 µUI/mL for TSH and 0.93–1.70 ng/dL for FT4.

Sex hormone levels [luteinizing hormone (LH), folliclestimulating hormone (FSH)] were assayed in serum using a clinical chemiluminescence immunoassay analyzer (CL-1000i, Mindray, Shenzhen, China). Units for these tests are µUI/mL. Anti-Müllerian hormone (AMH) levels were measured on serum samples using the Beckman Coulter AMH Gen II ELISA according to the manufacturer’s instructions. All assays were measured using an ELISA plate reader (Synergy HTX, BIO-TEK, Winooski, Vermont, USA). The minimal detectable AMH concentration with the AMH Gen II assay is 0.9 pmol/L (as advised by the manufacturer) and samples with undetectable levels were recorded as 0.9 pmol/L.

### 2.7. Oxidative Stress Biomarkers

Lipid peroxidation products were quantified in serum samples by the thiobarbituric acid (TBA) method [23]. Malondialdehyde (MDA) is formed as an end-lipid peroxidation product which reacts with the TBA reagent under acidic conditions to generate a pink colored product. Values were presented as nmol TBARS/mg proteins.

Protein carbonyl content, a biomarker of protein oxidation, was assayed with the method described by Levine et al. (1990) [24] using 2,4-dinitrophenylhydrazine (DNPH) prepared in 2 MHCl, 20% trichloro acetic acid (*w*/*v*), and 6 M guanidine hydrochloride. Results are expressed as nmol carbonyl/mg protein, using the extinction coefficient 22,000 M^−1^ cm^−1^.

### 2.8. Urinary Early Kidney Damage Biomarkers

Proteinuria was measured with the Bradford assay [25]. N-Acetyl-β-D-glucosaminidase (NAG) activity was quantified using a commercial kit [“N-Acetyl-β-D-glucosaminidase (NAG) assay kit”, Diazyme, Poway, CA, USA] following the manufacturer’s instructions. Neutrophil gelatinase-associated lipocalin (NGAL) was measured by commercial ELISA (“Human NGAL ELISA Kit 036CE”, BioPorto Diagnostics, Hellerup, Denmark), according to the manufacturer’s instructions; and for the quantification of Kidney injury molecule-1 (KIM-1), the kit “KIM-1 (human) ELISA Kit #ADI-900-226” (Enzo Life Sciences, Farmingdale, NY, USA) was used. Albumin was quantified using the “Human Albumin ELISA Quantitation Set E80-129” kit, and the “Human Transferrin ELISA Quantitation Set E80-128’’ kit was used to determine transferrin, both from Bethyl Laboratories, Montgomery, TX, USA. Both procedures require the “ELISA Starter Accessory kit E101’’ kit, which provides the necessary reagents for the determination of both proteins. All biomarkers values in humans were factored by urinary creatinine concentration with the objective of normalizing the effect of urine concentration [26]. The urinary creatinine required for the normalization of all biomarkers was measured using the commercial kit “Quantichrom creatinine assay kit” (BioAssay Systems, Haywar, CA, USA). 

In addition, these biomarkers do not have reference values established as “normal” or “physiological”, a battery of urinary samples from a control group consisting of 16 healthy women (non-working in any of the activities described in point 2.2. and non-residents in Marinaleda) with demographic, anthropometric and biochemical characteristics statistically similar to the women in the groups’ “farmers” and “NOE” (data not shown) was included.

### 2.9. Statistical Analysis

Descriptive statistics were generated for demographic parameters in the farmers group and NOE group. Outliers were identified using the Grubbs test [27]. Frequencies and percentages for all the categorical parameters were compared between both groups using Pearson’s chi-squared or Fisher’s exact test. In the case of continuous quantitative variables, firstly, it was studied whether the data in the groups followed a normal distribution, applying Shapiro–Wilk’s test. After that, an unpaired student’s t test was used to compare the mean values of the quantitative characteristics (demographic parameters, oxidative stress biomarkers, cholinesterase activity, biochemical and hematological parameters) between the farmers and NOE groups. On the other hand, the comparison of the urinary excretion of the biomarkers of kidney damage described in point 2.8. between the three groups was carried out with an ANOVA test coupled with a Scheffe post-hoc test (for normal and homoscedastic data, the latter evaluated with Levene’s test) or a Games-Howell post-hoc test (for normal and non-homoscedastic data); or a Kruskal-Wallis test (for non-normal data) with Bonferroni correction as post-hoc test. Finally, and with the aim of establishing the possible relationship between exposure to pesticides and the development of subclinical kidney damage in Marinaleda workers and residents, Spearman correlation studies (for non-normal data, evaluated with the Kolmogorov-Smirnov test) were carried out between excretion of the different biomarkers evaluated at all sampling times and the blood levels of cholinesterase, acetylcholinesterase, lipoperoxidase and protein oxidation.

The criterion for significance was set at *p* < 0.05. All the statistical analysis was performed using the IBM SPSS statistics software version 24.0 (International Business Machines, Armonk, NY, USA). Microsoft Office Excel 2016 and PowerPoint 2016 (Microsoft, Redmond, WA, USA) were used to create the artwork and illustrations presented.

## 3. Results

### 3.1. Characteristics of the Studied Population 

Characteristics of the study population are shown in Table 2. Twenty-two women farmers involved in recollection of vegetables and fruits were evaluated in comparison with the NOE group formed by 17 women who work in the canned factory in the same town at least in the last 10 years in order to ensure that they were not previously involved in farming activities. All the women were working in their jobs at least in the last 5 years. Farmers are working regularly along the year collecting the different kinds of crops depending *on* the time of year, their exposure is considered as chronic exposure. Nonsmokers were the majority of the women from both groups. Most of the participants in both groups are non- or sporadic (only in special events) alcohol consumers, only 22% and 36% were weekend alcohol consumers in farmers and NOE groups, respectively. 

Health status and neurological endpoints were assessed taking into account the self-reported symptoms collected through the epidemiological questionnaire. A variety of neurological symptoms, including headache (25%) and dizziness (25%), gastrointestinal and respiratory symptoms (35%, respectively) have been reported in women farmers. These symptoms were significant as compared to NOE participants. The health status was assessed along the monitoring period through three more questionnaires. There were no significant differences in neurological, gastrointestinal or respiratory symptoms collected between the same participants and only 5% of farmers informed about a weight gain after the first sample collection.

### 3.2. Effects on Cholinesterase’s Activities

**Figure 1:**
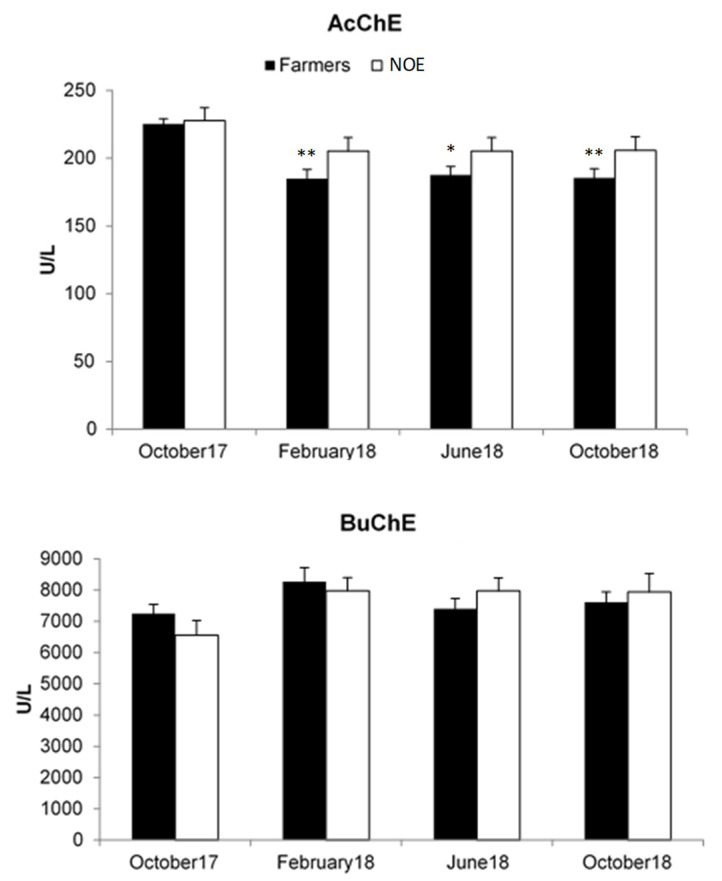
Activities of AChE in erythrocyte and BuChE in serum evaluated at different sampling times. Data are expressed as the mean ± standard error of the mean (SEM). * *p* < 0.05; ** *p* < 0.01 versus farmers (October17). AChE: acetylcholinesterase; BuChE: butyrylcholinesterase; NOE: non-occupational exposed.

The activities of AChE in erythrocyte and BuChE in serum have been estimated and represented in Figure 1. No statistically significant alterations in cholinesterase activity, neither AChE nor BuChE, were found in women farmers when compared to the NOE women group. However, it can be observed that AChE activity was slightly lower in farmers than in NOE group in the last three monitoring dates (February, June and October 2018). Thus, when comparing the AChE activity detected in the farmers group in these three dates there was a statistically significant decrease (≈18%) (*p* < 0.05 and *p* < 0.01) in relation with those detected in the first sampling date (October 2017).

### 3.3. Effects on Hematological and Biochemical Parameters

No significant differences were found in results from the hemograms when comparing farmers and NOE groups along all of the monitoring period. 

Biochemical parameters are shown in Table 3. Women farmers presented a significant increase in serum levels of glucose when compared to NOE groups in 17 October (*p* < 0.05) and 18 October (*p* < 0.001). On the other hand, the levels of total proteins in women farmers were significantly reduced in relation to NOE women groups only in 18 February (*p* < 0.001). Regarding lipid profile, a very significant increase in HDL (*p* < 0.01 and *p* < 0.001) along all the studied monitoring periods were observed in farmer women as compared with NOE groups. Interestingly, a decrease in LDL (18 June and 18 October) and triglycerides (18 February and 18 June) (*p* < 0.01 and *p* < 0.001) was also observed, being the NOE group the population that presents a significant increase in lipid parameters even with values above of the reference values, suggesting abnormal accumulation of lipids. The liver function biomarkers assessed, AST, ALT and LDH didn’t show significant differences between both groups of population (farmers and NOE) in none of the four periods studied. Even though serum urea and creatinine increased in different study periods in the farmers group compared to the NOE group, in no case they exceeded the reference values, so the results showed a preserved kidney function for both groups.

### 3.4. Effects on Hormonal Analysis

Results for hormonal analysis (TSH, FT4, LH, FSH and AMH) are shown in Table 4. There is a great variability in results of all of these parameters in both studied groups (farmers and NOE) and between the different periods of the study (17 October, 18 February, 18 June and 18 October). Compared with the NOE group, only levels of FT4 and LH were significantly decreased in farmer women in June and October 2018, respectively. Levels of FT4 and TSH were within the reference values in both populations (farmers and NOE). Furthermore, levels of AMH in three monitoring periods measured were within enough levels of ovarian reserve.

### 3.5. Effects on Lipid and Protein Oxidation

**Figure 2:**
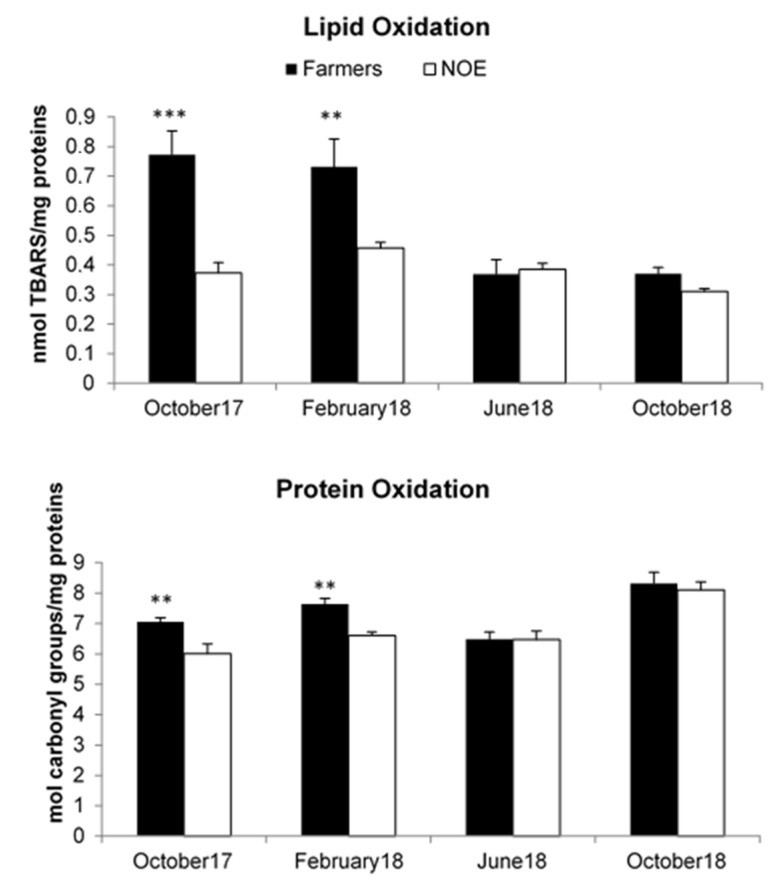
Activity of the biomarkers of oxidative stress at different sampling times. Data are expressed as the mean ± SEM; ** *p* < 0.01; *** *p* < 0.001 versus NOE group. NOE: non-occupational exposed.

The activity of the biomarkers of oxidative stress studied in farmers and NOE women at the four study periods (October 2017, February 2018, June 2018 and October 2018) are shown in Figure 2. As compared to NOE groups, women farmers had significantly higher levels of TBARS and carbonyl groups in the two first sampling (17 October and 18 February) (*p* < 0.01 and *p* < 0.001). In the last two samples it was observed a decrease in levels of TBARS and carbonyl groups in women farmers without significant differences in comparison to the values of NOE. There were no significant differences in levels of TBARS and carbonyl groups in the NOE group along the four sampling periods.

### 3.6. Effects on Early Renal Function Biomarkers

**Figure 3:**
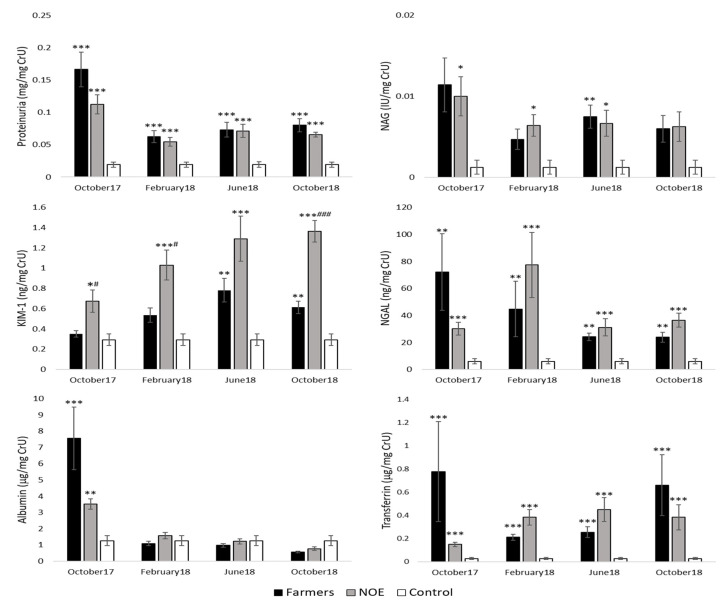
Urinary levels of the biomarkers of early kidney damage evaluated at different sampling times. Data are expressed as the mean ± SEM. * *p* < 0.05; ** *p* < 0.01; *** *p* < 0.001 versus control group. # *p* < 0.05; ### *p* < 0.001 versus farmers group. CrU: urinary creatinine; KIM-1: kidney injury molecule 1; NAG: N-acetyl-β-D-glucosaminidase; NGAL: neutrophil gelatinase-associated lipocalin; NOE: non-occupational exposed.

Urinary levels of the biomarkers of early kidney damage are shown in Figure 3. As can be observed, all the evaluated biomarkers presented at least at some of the sampling times high levels in their excretion with respect to the control group, on many occasions with high statistical significance (*p* < 0.001). Even though, in general, no statistically significant differences were detected between the farmers and NOE groups, a greater excretion of KIM-1 (in February and October 2018) was observed in the second group.

In order to study if there was a relationship between kidney damage and pesticide exposure, a correlation study was carried out between kidney damage biomarkers and pesticide exposure (measured by AChE and BuChE) (Table 5). Our results showed that AChE, a biomarker of chronic and low-intensity exposures, correlated with a higher number of biomarkers of kidney damage in the farmers group compared to the NOE group. These data seem to show a relationship between exposure to pesticides (farmers group) and kidney damage. However, BuChE, a biomarker of acute exposure, in both groups correlates with two of the biomarkers of kidney damage evaluated. These data seem consistent with those obtained from BuChE, in which no significant differences were observed throughout the four samplings between the farmers and NOE groups.

## 4. Discussion

This work evaluated in two populations of rural women the biochemical, hematological, hepatic, hormonal and renal alterations induced by indirect exposure to ChE inhibitor pesticides, as well as the implication of oxidative stress in these effects. The study was carried out in Marinaleda (Seville). This town is surrounded by the fields where most of the population work, either sowing, applying pesticides or collecting the fruits. One group consisting of women farmers indirectly exposed to pesticides during harvesting and the other one formed by women non-occupationally exposed to these compounds due to their work in the canned factory of the town. Considering the low number of studies investigating the health of harvest farmers, especially women, our study provides important data on the effects associated with this indirect exposure to pesticides in this population group in comparison with the NOE group, and about the influence of the rural environment when comparing these two rural groups with a non-rural control group. The strength of the present study lies in the fact that all the parameters studied were assessed four times along the study in order to relate the observed effects to a chronic exposure.

During the sampling, farmer women were in the middle of the harvest time, collecting different cultures along the study. Our data showed that most of these women (88.9%) were chronically and indirectly exposed to pesticides during more than 10 years. All of them reported the use of PPE but only gloves because none of them reported the use of masks or glasses. Similar response was obtained in the NOE group about using PPE in their work in the factory. No significant differences were observed in BuChE activities along the four sampling when farmer and NOE are compared. However, AChE activity was found to be lower (10%, 8% and 10%) in the second, third and fourth sampling, in women farmers as compared to the NOE group, but was not found to be statistically significant. The inhibition observed in AChE activities along the sampling period, is very far from the Biological Exposure Index (BEI) that establish a 70% of AcChE activity an individual’s baseline as a reference value for exposure control, what means a 30% of decreased in the enzymatic activity (INSST, 2019). Even though both enzymatic activities are markers of early biologic effects related to OPs and carbamates exposure, AChE inhibition is more sensitive and preferred than BuChE, since it reflects the biological effects on the nervous system and shows a lower recovery rate, representing the inhibition of the neural AChE in a more realistic manner [1]. The inhibition of cholinesterases activities has been associated with cholinergic dysfunctions in pesticides-exposed workers [2], mainly in pesticide sprayers who are directly exposed to these compounds. Thus, significant reduction in the enzymatic activity (17%−26%) was observed in pesticide sprayers and in women plucking leaves in tea plantations from India [2,7,28]. Similar results were reported by Cestonaro et al. (2020) [1] in farmers from Brazil with a decreased enzymatic activity of 21% for AChE in comparison with NOE group. On the other hand, and more in agreement with our results, Vikkey et al. (2017) [29] observed that 88% of the cotton farmers from Benin displayed less than 20% AChE inhibition. Some authors have concluded that despite AChE activity being used to biomonitor exposure to pesticides in the human population, in some cases no differences in this enzymatic activity are found among exposed populations and NOE groups [8,30].

Another interesting finding of this study is that provided by the alteration of the biochemical parameters in the population groups studied. Even though weakly, AChE was significantly and inversely associated with glucose, showing that the chronic exposure to pesticides can also contribute to the increase in glucose levels [1]. Our results showed a significant increase in glucose levels in women farmers in comparison with the NOE group in two of the four sampling dates. However, despite the chronic exposure of this group, the measured levels of glucose did not represent a real hyperglycemia, probably since the pesticides employed are not only OPs and carbamates and that the exposure to these compounds is indirect. Further, our study also showed a significant increase in HDL levels as compared to NOE group, however, a trend of decreased in the levels of lipid profile including cholesterol, triglycerides and LDL comparing to NOE group indicating that the indirect exposure to pesticides in this population of women does not lead to an abnormal accumulation of lipid, altered metabolism, hepatic dysfunction or cardiac problems as it has been observed in previous studies in pesticide sprayers [2]. The long-term exposure to pesticides may increase the levels of AST, ALT and LDH, causing damage to liver function. In the present study no significant differences were observed in these parameters when comparing both groups (farmers vs. NOE). This fact with the significant increase in HDL levels lead us to conclude that there is no liver damage in this indirectly exposed population. 

Some pesticides may interfere with the female hormonal function, which may lead to negative effects on the reproductive system through disruption of the hormonal balance necessary for proper functioning [14]. In the present study slightly lower levels in FT4 and LH were observed in farmers in comparison with the NOE group, but only in June and October 18 were statistically significant, respectively. Despite these differences, levels of FT4 both in farmer and NOE groups along the study were all within the normal reference values. Some authors have observed a relationship between significant increases in FT4 levels and pesticide exposure, suggesting that occupational exposure to pesticides may affect thyroid function [3,20]. In relation with LH levels, most of the values detected in both groups are higher than 25 U/L considered as a menopause indicator in elder women or a signal of ovarian malfunction or an early menopause in younger women. 

Kidney damage from pesticides exposure has been referenced in various studies [11,31,32,33,34], however, most of them have been carried out in conditions in which chronic heat stress and dehydration have also been considered as etiopathogenic factors of renal damage. This could justify that in our study, kidney damage, measured by an increase in creatinine, was not evidenced since the study groups did not suffer from these conditions. Furthermore, the increase in creatinine is only evidenced when renal function has decreased by 50% [35], which is not the circumstance of our study. During the last two decades, new biomarkers have been identified that detect kidney damage independently of serum creatinine, even under subclinical circumstances. These biomarkers (KIM-1, NGAL, NAG, etc.), have demonstrated their ability to diagnose early kidney damage, give information on the type of injury and progression of damage. It has been proven that they are metabolic components or derivatives, degradation compounds or remnants of them that appear in the urine as a result of damage to kidney structures [36,37,38,39]. In this study we have identified different urinary biomarkers (protein, albumin, transferrin, KIM-1, NAG and NGAL), which are related to a subclinical tubular alteration [40,41,42]. The importance of this finding is that repeated tubular damage could progress to chronic kidney disease [43]. In fact, chronic kidney disease of unknown etiology is a global epidemic whose cause has not identified a single factor, but many factors that can contribute to the etiology of the disease, including exposure to agrochemicals, particularly glyphosate and paraquat, are likely compound factors and may be the primary factors [34,44]. This is the first study to alert to the possibility of subclinical kidney damage in a rural setting with indirect exposure to pesticides.

On the other hand, this study presented results regarding the levels of intoxication biomarkers such as BuChE and AChE. These enzymes are important markers for the investigation of organophosphate pesticide poisonings after acute and chronic exposures, respectively [45]. Therefore, these data confirm the real impact of exposure to these compounds on kidney function. Another of the relevant findings in our study is that subclinical kidney damage appears both in women indirectly exposed to pesticides (farmers) and those exposed in a non-occupational way (NOE). These results suggest that the effect of pesticides could affect a general population in a rural environment based on agriculture and therefore, possible preventive measures should be extended to the entire population.

Oxidative stress as a possible mechanism of toxicity for pesticides has become a focus of toxicological research because it is considered as a critical pathophysiological mechanism in different human pathologies associated with pesticide exposure [30]. Oxidative damage may result in cellular adaptation, damage to cellular lipids, DNA, proteins and/or cellular death [3]. In the present study, lipid peroxidation and protein oxidation as biomarkers of oxidative stress were investigated among women farmers who are indirectly exposed to a mixture of pesticides and women who are not occupationally exposed to these compounds. MDA and TBARS as a direct measure of MDA have been used as a biomarker of lipid peroxidation and is one of the most reliable markers to determine oxidative stress in clinical situations [10,30]. Several studies reported increased MDA concentrations in applicators, farmers and sprayers exposed to different pesticides [2,4,8,10]. In this study MDA levels were higher in women farmers than in the NOE group, but only in two of the sampling dates (17 October and 18 February). Similarly, levels of protein oxidation measures as nmol of carbonyl groups were higher in farmers than in NOE group in samples from 17 October and 18 February. It has been demonstrated that oxidative stress is the main mechanism of acute intoxication from some pesticides, either individually or in a mixture, although the underlying molecular mechanisms are not clear [10,46]. This fact could explain the recovery observed in both in the farmer’s biomarkers to the control levels along the study.

Despite these findings, the main limitation of the study is the low sample size. On the other hand, the results cannot be extrapolated to the general population, since the study has been carried out in women in fertile age from a rural setting. Thus, further research is necessary to strongly address the issue of pesticide toxicity in both genders’ farmers and in a greater age range.

## 5. Conclusions

The present study showed for the first time that the indirect chronic exposure to pesticides lead to a subclinical tubular damage in a women rural setting that could progress to chronic kidney disease. In addition, the oxidative stress biomarkers measured were higher in the women farmer group with respect to NOE group at the beginning of the study, showing a recovery to control levels with time as indicative of acute intoxication. However, no significant differences were detected in cholinesterase activities, the classical biomarkers of pesticide exposure, between both groups studied. In view of the results obtained for this investigation, it may be concluded that farmers are exposed to a mixture of pesticides beyond organophosphates and carbamates, so it is necessary to implement new biomarkers in order to improve the monitoring of the health status in farmers to protect properly this vulnerable population.

## Figures and Tables

**Table 1:** Chronogram of sampling, crops collected and the pesticides most used along the whole study.

Date of Sampling (Day/Month/Year)	Crop Collected in the Previous Three Months	Pesticides Applied	Target Pest
5 October 2017	Pepper Garlic Artichoke	Pendimethalin	Herbicide
		Fluazifop-P-butyl	Herbicide
		λ-Cyhalothrin	Insecticide
8 February 2018	Garlic Artichoke Broccoli Green beans Olives Wheat	Bromoxynil	Herbicide
		Fluazifop-P-butyl	Herbicide
		Glyphosate	Herbicide
		Dimethylamine	Herbicide
		Diflufenican	Herbicide
		Chlortoluron	Herbicide
		Tritosulfuron	Herbicide
		Imidacloprid	Insecticide
		λ-Cyhalothrin	Insecticide
		Mancozeb	Fungicide
		Azoxystrobin	Fungicide
		Copper oxychloride	Fungicide
6 June 2018	Pepper Garlic Artichoke Broccoli Green beans Olives Wheat Cotton Zucchini Chickpeas Sunflowers Chamomile	Cycloxydim	Herbicide
		Fluazifop-P-butyl	Herbicide
		Pendimethalin	Herbicide
		Glyphosate	Herbicide
		Imazamox	Herbicide
		Dimethylamine	Herbicide
		Fluometuron	Herbicide
		Ethofumesate	Herbicide
		Fluroxypyr	Herbicide
		Napropamide	Herbicide
		Tritosulfuron	Herbicide
		Bromoxynil	Herbicide
		Pinoxaden	Herbicide
		λ-Cyhalothrin	Insecticide
		Chlorpyrifos	Insecticide
		Deltamethrin	Insecticide
		Betacyfluthrin	Insecticide
		Propanocarb	Fungicide
		Chlorthanolil	Fungicide
		Copper oxychloride	Fungicide
		Azoxystrobin	Fungicide
		Tebuconazole	Fungicide
10 October 2018	Pepper Garlic Artichoke	Pendimethalin	Herbicide
		Fluazifop-P-butyl	Herbicide
		λ-Cyhalothrin	Insecticide

**Table 2:** General characteristics of farmers and non-occupational exposed (NOE) groups. n.c.: not calculable; PPE: personal protective equipment. Data are expressed as number of patients [*n*, (%)].

	Farmers (*n* = 22)	NOE (*n* = 17)	*p*-Value
Age			0.175
18–28 years	3 (13.6)	3 (17.6)	
29–38 years	3 (13.6)	6 (35.3)	
39–45 years	16 (72.7)	8 (47.1)	
Smoking habits			1.000
Smokers	8 (36.4)	6 (35.3)	
Non-smokers	14 (63.6)	11 (64.7)	
Alcohol consumption			0.015
Non-consumer	10 (45.5)	11 (64.7)	
Sporadic	8 (36.4)	0 (0)	
Weekend	4 (18.2)	6 (35.3)	
Number of years working at that job			0.006
<5	2 (9.1)	0 (0)	
5–10	0 (0)	5 (29.4)	
>10	20 (90.9)	12 (70.6)	
Use of PPE			0.184
Yes	22 (100)	15 (88.2)	
No	0 (0)	2 (11.8)	
Type of PPE			
Mask	0 (0)	0 (0)	n.c.
Gloves	22 (100)	15 (88.2)	0.184
Glasses	0 (0)	0 (0)	n.c.

**Table 3:** Effects on biochemical and on liver and renal profile in farmers and in non-occupational exposed (NOE) groups. Data are expressed as the mean ± SEM. * *p* < 0.05; ** *p* < 0.01; *** *p* < 0.001 versus NOE group. ALT: alanine transaminase; AST: aspartate aminotransferase; HDL: high-density lipoprotein; LDH: lactate dehydrogenase; LDL: low-density lipoprotein.

	October 2017		February 2018		June 2018		October 2018		Normal Range
	Farmers	NOE	Farmers	NOE	Farmers	NOE	Farmers	NOE	
Glucose (mg/dL)	91.6 ± 2.8 *	81.7 ± 1.7	79.0 ± 3.1	76.7 ± 1.6	75.8 ± 1.5	77.4 ± 1.6	82.1 ± 4.9 ***	61.1 ± 2.0	75–110
Total proteins (g/dL)	7.0 ± 0.1	6.9 ± 0.1	7.5 ± 0.1	7.4 ± 0.0	7.0 ± 0.1 ***	7.4 ± 0.0	7.6 ± 0.1	7.4 ± 0.1	6.5–8.0
Lipid profile									
Total cholesterol (mg/dL)	187.6 ± 3.4	174.7 ± 12.1	193.3 ± 4.8	199.9 ± 13.8	186.6 ± 4.3	200.4 ± 13.7	185.3 ± 6.6	218.3 ± 17.1	90–220
HDL (mg/dL)	55.0 ± 2.7 **	43.3 ± 2.6	59.3 ± 2.1 ***	42.4 ± 2.8	60.2 ± 2.3 ***	42.3 ± 2.8	58.0 ± 2.3 **	46.8 ± 1.6	35–65
LDL (mg/dL)	119.4 ± 3.2	116.8 ± 10.0	125.7 ± 3.9	144.1 ± 11.8	105.3 ± 3.4 **	144.2 ± 11.8	100.4 ± 5.1 **	142.1 ± 11.7	<129
Triglycerides (mg/dL)	137.5 ± 11.2	128.2 ± 10.0	135.9 ± 9.1 **	198.1 ± 14.8	106.7 ± 8.2 ***	198.2 ± 14.7	136.4 ± 9.6	148.9 ± 20.1	50–200
Hepatic function biomarkers									
LDH (U/L)	401.8 ± 11.6	376.7 ± 15.9	371.0 ± 10.7	373.3 ± 14.4	355.7 ± 10.1	373.9 ± 14.3	412.7 ± 11.0	437.7 ± 13.3	230–460
AST (U/L)	20.6 ± 2.4	19.1 ± 1.5	23.0 ± 2.3	19.3 ± 0.9	17.2 ± 1.3	19.4 ± 1.0	18.3 ± 1.1	21.0 ± 1.1	10–37
ALT (U/L)	20.1 ± 2.9	18.3 ± 3.2	23.7 ± 3.6	18.4 ± 2.9	17.7 ± 1.9	18.8 ± 2.8	15.6 ± 1.5	14.6 ± 1.1	10–40
Renal function biomarkers									
Urea (mg/dL)	33.8 ± 1.9 ***	22.0 ± 1.2	30.1 ± 0.7 ***	21.2 ± 0.6	33.5 ± 1.7 ***	21.6 ± 0.7	34.0 ± 1.2 ***	23.9 ± 1.4	15–50
Creatinine (mg/dL)	0.8 ± 0.0 ***	0.7 ± 0.0	0.7 ± 0.0 *	0.6 ± 0.0	0.7 ± 0.0	0.6 ± 0.0	0.7 ± 0.2	0.7 ± 0.0	0.6–1.2

**Table 4:** Effects on hormonal levels in farmer women and in non-occupational exposed (NOE) groups. Data are expressed as the mean ± SEM. ** *p* < 0.01; *** *p* < 0.001 versus NOE group. AMH: anti-Müllerian hormone; FSH: follicle stimulating hormone; FT4: free thyroxine; LH: luteinizing hormone; n.m.: not measured; TSH: thyroid-stimulating hormone.

	October 2017		February 2018		June 2018		October 2018		Normal Range
	Farmers	NOE	Farmers	NOE	Farmers	NOE	Farmers	NOE	
TSH (mIU/L)	1.33 ± 0.16	1.86 ± 0.27	1.87 ± 0.17	1.61 ± 0.16	1.41 ± 0.12	1.62 ± 0.17	1.88 ± 0.18	2.70 ± 0.78	0.27–5.50
FT4 (ng/dL)	1.16 ± 0.01	1.27 ± 0.06	1.18 ± 0.03	1.22 ± 0.07	1.04 ± 0.02 **	1.23 ± 0.07	1.19 ± 0.02	1.23 ± 0.04	0.93–1.70
LH (U/L)	24.2 ± 6.8	38.7 ± 11.0	31.5 ± 10.1	56.3 ± 14.6	25.3 ± 7.3	55.6 ± 14.5	18.5 ± 6.6 ***	97.8 ± 12.4	<25 U/L
FSH (U/L)	19.1 ± 4.7	51.8 ± 28.4	21.6 ± 5.4	29.3 ± 5.5	17.0 ± 4.1	29.2 ± 5.5	12.8 ± 3.2	42.6 ± 4.3	3.8–8.8 U/L (follicular phase); 1.8–5.1 U/L (luteal phase); 4.5–22.5 U/L (menstrual cycle)
AMH (ng/mL)	n.m.	n.m.	1.0 ± 0.3	1.1 ± 0.3	0.9 ± 0.2	1.1 ± 0.3	1.1 ± 0.2	1.4 ± 0.7	0.7–2.3 ng/mL enough levels of ovarian reserve

**Table 5:** Results of the correlation study carried out between the urinary biomarkers evaluated and blood parameters of exposure to pesticides. Data are expressed as Spearman’s correlation coefficient (ρ). Significance of the correlation: * *p* < 0.05; ** *p* < 0.01; ***p < 0.001. AChE: acetylcholinesterase; BuChE: butyrylcholinesterase; KIM-1: kidney injury molecule 1; NAG: N-acetyl-β-D-glucosaminidase; NGAL: neutrophil gelatinase-associated lipocalin; NOE: non-occupational exposed.

Farmers		Proteinuria	Urinary NAG	Urinary KIM-1	Urinary NGAL	Urinary Albumin	Urinary Transferrin
Exposure to pesticides	Blood BuChE	−0.29 *	0.14	0.23	0.14	−0.26 *	−0.18
	Blood AChE	0.08	0.01	−0.43 ***	−0.33 **	0.38 **	−0.27 *
Oxidative stress	Blood lipoperoxidase	−0.09	−0.03	−0.09	−0.02	0.21	−0.47 ***
	Blood proteins oxidation	−0.22	−0.15	0.06	0.14	−0.09	0.12
NOE		Proteinuria	Urinary NAG	Urinary KIM-1	Urinary NGAL	Urinary albumin	Urinary transferrin
Exposure to pesticides	Blood BuChE	−0.14	−0.20	0.61 ***	−0.16	−0.36 ***	0.13
	Blood AChE	0.05	−0.08	−0.03	−0.36 **	0.04	−0.35 **
Oxidative stress	Blood lipoperoxidase	0.09	−0.16	0.08	0.05	0.20	0.08
	Blood proteins oxidation	−0.08	−0.02	0.09	−0.14	−0.27*	0.17

## Data Availability

The data presented in this study are available on request from the corresponding author.
